# Assessing Open Science practices in physical activity behaviour change intervention evaluations

**DOI:** 10.1136/bmjsem-2021-001282

**Published:** 2022-05-23

**Authors:** Emma Norris, Isra Sulevani, Ailbhe N Finnerty, Oscar Castro

**Affiliations:** 1 Department of Health Sciences, Brunel University London, Uxbridge, Middlesex, UK; 2 Centre for Behaviour Change, University College London, London, UK; 3 Future Health Technologies, Singapore-ETH Centre, Campus for Research Excellence And Technological Enterprise, Singapore

**Keywords:** open science, reproducibility, transparency, meta-research, physical activity, behaviour change

## Abstract

**Objectives:**

Concerns on the lack of reproducibility and transparency in science have led to a range of research practice reforms, broadly referred to as ‘Open Science’. The extent that physical activity interventions are embedding Open Science practices is currently unknown. In this study, we randomly sampled 100 reports of recent physical activity randomised controlled trial behaviour change interventions to estimate the prevalence of Open Science practices.

**Methods:**

One hundred reports of randomised controlled trial physical activity behaviour change interventions published between 2018 and 2021 were identified, as used within the Human Behaviour-Change Project. Open Science practices were coded in identified reports, including: study pre-registration, protocol sharing, data, materials and analysis scripts sharing, replication of a previous study, open access publication, funding sources and conflict of interest statements. Coding was performed by two independent researchers, with inter-rater reliability calculated using Krippendorff’s alpha.

**Results:**

78 of the 100 reports provided details of study pre-registration and 41% provided evidence of a published protocol. 4% provided accessible open data, 8% provided open materials and 1% provided open analysis scripts. 73% of reports were published as open access and no studies were described as replication attempts. 93% of reports declared their sources of funding and 88% provided conflicts of interest statements. A Krippendorff’s alpha of 0.73 was obtained across all coding.

**Conclusion:**

Open data, materials, analysis and replication attempts are currently rare in physical activity behaviour change intervention reports, whereas funding source and conflict of interest declarations are common. Future physical activity research should increase the reproducibility of their methods and results by incorporating more Open Science practices.

**WHAT IS ALREADY KNOWN ON THIS TOPIC**

- Open Science practices support research being more transparent and reproducible.
- Assessment of Open Science practices in physical activity research is limited.

**WHAT THIS STUDY ADDS**

- We reviewed Open Science practices within 100 reports of physical activity behaviour change intervention randomised controlled trials.
- Open data, materials, analysis and replication attempts are currently rare in physical activity behaviour change intervention research.

**HOW THIS STUDY MIGHT AFFECT RESEARCH, PRACTICE AND/OR POLICY**

- Our study draws attention to the practical solutions and resources that are most needed to promote transparency, reproducibility and accessibility in future physical activity research.

## Introduction

Across scientific research, there is an increased awareness of highly prevalent problematic research practices, often referred to as questionable research practices,^1^ such as *p-*hacking: mining data for significant results^2 3^ and Hypothesising After the Results are Known (or ‘HARKing’).^4^ Open Science is an umbrella term of research behaviours intending to reduce these questionable research practices.^5–8^ Open Science research practices can be applied across the whole research process: from conception to publication.^7–9^ At research conception, pre-registrations provide time-stamped evidence of study hypotheses, methods and analysis plans,^10 11^ with these details made publicly available through online repositories such as Open Science Framework.^12^ In contrast to research protocols that specify research details but may be published before or after the study is in-progress or even completed,^13^ pre-registrations are completed and published prior to data collection to minimise biases.^14^ Open data, open materials (including questionnaires and intervention materials used) and open analysis scripts help make the processes and outputs of research more transparent, accessible and shareable.^15^ At publication, open access publishing makes reporting of research available to anyone at no cost to the reader.^16^

Questionable research practices are likely rife in physical activity, sport and exercise medicine research.^17 18^ A recent study assessed the prevalence of questionable research practices within sport and exercise medicine research, including 129 studies published in leading sports medicine journals in 2019.^19^ Their analysis found that 82.2% of all reported hypotheses, and 70.8% of primary hypotheses, were supported by study results identified as implausibly high.^19^ Meta research has assessed Open Science practices in domains related to physical activity, behaviour change and life sciences.^20^ A recent study exploring 250 psychology studies of varying study designs published between 2014 and 2017 found that while open access publication was relatively common (65%), sharing of open materials (14%), data (2%) and analysis scripts (1%), as well as pre-specification of research plans via pre-registration (3%) and study protocols (0%) were low.^21^ In addition, transparency of reporting was inconsistent for funding statements (62%) and conflict of interest disclosure statements (39%).^21^ Meta-science studies have also assessed these Open Science practices within smoking cessation behaviour change research,^22^ social sciences,^20^ biomedicine^23^ and biostatistics.^24^

However, to our knowledge, no study has evaluated the extent to which Open Science practices are used within physical activity research. Gaining a better understanding of these practices could inform future recommendations and policy development to promote open, transparent science within the physical activity field and to reduce the threat of questionable research practices. Therefore, the aim of this study was to assess Open Science practices within physical activity behaviour change intervention randomised controlled trial (RCT) reports assessing moderate-to-vigorous physical activity outcomes.

## Methods

### Study design

This was a retrospective observational study with a cross-sectional design. Sampling units were individual physical activity behaviour change intervention reports. This study applied an established methodology used to assess Open Science practices in smoking cessation interventions,^22^ psychological sciences^21^ and social sciences.^20^ This study was pre-registered on the Open Science Framework.^25^ All deviations from this protocol are explicitly acknowledged in online supplemental file 1. Deviations included adding an additional item to specify whether a declared study pre-registration was registered ahead of data collection, or whether it was actually retrospectively registered after data collection had commenced, as well as adding ‘funded by a non-profit’ options within funding source and conflict of interest assessment items.

10.1136/bmjsem-2021-001282.supp1 Supplementary data

### Search strategy

**Figure 1:**
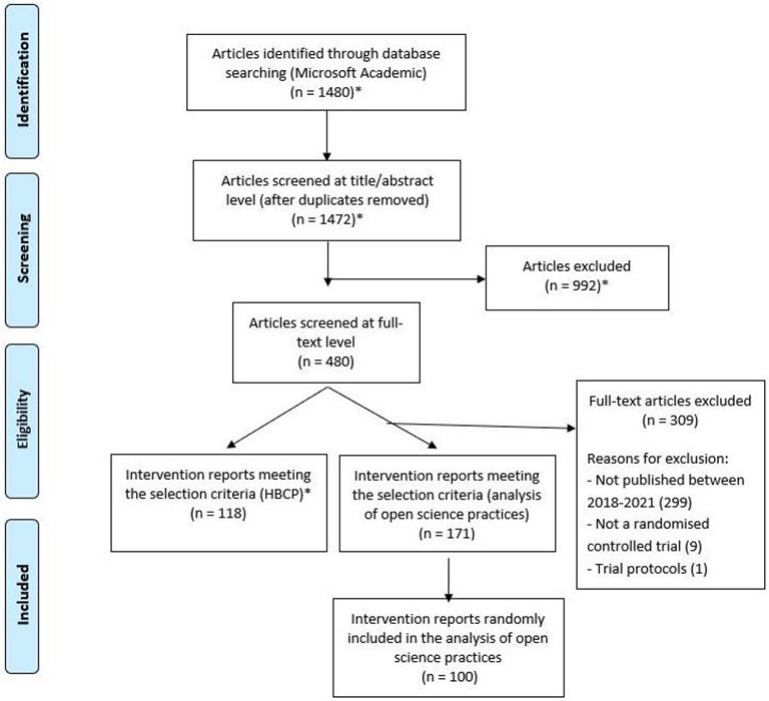
Flow diagram for the physical activity behaviour change intervention reports included in the analysis of Open Science practices. * Steps performed as part of the Human Behaviour-Change Project (HBCP) and involving two independent reviewers.

All papers included in this study were reports of physical activity behaviour change interventions, evaluated via RCTs. These reports were identified for inclusion within the Human Behaviour-Change Project (HBCP), which is developing an artificial intelligence system to extract information from published intervention studies and make recommendations for real-world practice and future research.^26 27^ The selection criteria for the HBCP are comparable to the one used for the present study (ie, both projects have a broad scope and aim to identify a subsample of reports describing RCTs of physical activity behaviour change interventions). Therefore, we used the same pool of articles remaining after the HBCP’s title and abstract screening (see figure 1 for a complete overview). Physical activity behaviour change intervention reports were identified in the HBCP using Microsoft Academic, one of the biggest, most comprehensive bibliographic databases of scientific literature.^28^ The search strategy was performed on 20 January 2021 and included the terms ‘MVPA or moderate-to-vigorous physical activity or MPA or VPA or moderate physical activity or vigorous physical activity or strenuous physical activity or hard physical activity’, with studies additionally filtered using the RCT classifier within Microsoft Academic. The terms were identified through a scoping search in which one of the study authors (OC) manually scanned the terms used in 20 physical activity behaviour change intervention reports.

Inclusion criteria were reports describing RCTs of physical activity behaviour change interventions and published between 2018 and 2021. The rationale for the recency of these included papers is to best represent current Open Science practices, given the relatively recent nature of Open Science practices.^1^ In addition, we focused on RCTs only due to their recognition as ‘gold-standard’ for studying intervention effectiveness.^26^ Exclusion criteria were trial protocols, conference submissions, abstract-only entries, qualitative research and economic or process evaluations. Full texts of identified papers within the HBCP were screened by one researcher (EN) to double-check relevance against inclusion and exclusion criteria, with piloting of the screening strategy by two authors (EN and OC). Of the 171 reports remaining after applying these criteria, 100 reports were selected due to time and resource constraints using the Calculator Soup Random Number Generator.^29^

### Measures

Article characteristics recorded were: (i) author name, (ii) publication year and (iii) country of the corresponding author. Open Science practices were assessed by recording presence of the following in included reports: (i) *Pre-registration:* whether pre-registration was reported as carried out, where the pre-registration was hosted (eg, Open Science Framework, ClinicalTrials.gov), whether it could be accessed, what aspects of the study (hypotheses, methods and analysis plans) were pre-registered and whether the pre-registration was logged prospectively (prior to data collection commencing) or retrospectively (after data collection had commenced)^30^; (ii) *Protocol sharing:* whether a protocol was reported as published and what aspects of the study (hypotheses, methods and analysis plans) were included in the protocol; (iii) *Data sharing*: whether data were reported as available, where it was available (eg, online repository such as Open Science Framework, on request from authors, as a journal supplementary file), whether the data were downloadable and accessible, whether data files were clearly documented and the extent that data reported were sufficient to allow replication of study findings; (iv) *Materials sharing:* whether study materials were reported as available, where they were available (eg, online repository such as Open Science Framework, on request from authors, as a journal supplementary file) and whether the materials were downloadable and accessible; (v) *Analysis script-sharing:* whether analysis scripts were reported as available, where they were available (eg, online repository such as Open Science Framework, on request from authors, as a journal supplementary file) and whether the analysis scripts were downloadable and accessible; (vi) *Replication of a previous study:* whether the study was described as being a replication attempt of a previous study; (vii) *Open access publication:* whether the study was published as open access, assessed via the open access button website^31^ which harvests deposited publication from 1000s of academic institutions^32^; (viii) *Funding sources:* whether funding sources were declared and if research was funded by public organisations (such as research councils or charities), pharmaceutical, activity-related or other companies; and (ix) *Conflicts of interest:* whether conflicts of interest were declared and whether conflicts were with public organisations (such as research councils or charities), pharmaceutical, activity-related or other companies. The journal impact factor of identified papers was intentionally not assessed to evaluate papers, due to well-documented issues with manipulation and inflation of these figures.^33 34^ All measured variables are shown in table 1.

**Table 1:** Measured characteristics within identified physical activity behaviour change intervention reports

Variables	Coder questions	Response options
**Article characteristics**		
**Coder instructions:** Check the institutional affiliation of the corresponding author. If there are multiple corresponding authors, choose the first. If no corresponding author is identified, choose the first. If there are multiple affiliations for the selected author, choose the first.		
Country	Which country is the corresponding author based in according to their affiliated?	(List countries) / Unclear / Other
**Pre-registration**		
**Definitions:** ‘Pre-registration’ refers to the specification of important aspects of the study (typically hypotheses, methods and/or analysis plan) prior to commencement of the study.		
**Coder instructions:** Check specific sections in the paper where these files might be located, for example, supplementary materials, appendices, author notes, methods and results sections. Search for ‘registration’.		
Pre-registration statement	Does the article state whether or not the study (or some aspect of the study) was pre-registered?	Yes – the statement says that there was a pre-registration / Yes – the statement says that there was NO pre-registration / No – there is no pre-registration statement / Other*
Pre-registration method	Where does the article indicate the pre-registration is located?	Open Science Framework / AsPredicted / ClinicalTrials.gov / AEA Trial Registry / EGAP Registry / Registered Report / Other*
Pre-registration accessible	Can you access and open the pre-registration	Yes / No / Other*
Pre-registration content	What aspects of the study appear to be pre-registered? (select all that apply)	Hypotheses Methods Analysis Plan Other*
**Protocol sharing**		
**Definition:** ‘Protocol’ refers to a document containing details about the study design, methods and analysis plan. It may or may not be pre-registered.		
**Coder instructions:** Search the article for the phrase ‘protocol’ and assess whether a link is provided to a protocol document.		
Protocol availability	Does the article link to an accessible protocol?	Yes / No / Other*
Protocol content	What aspects of the study appear to be included in the protocol? (select all that apply)	Hypotheses Methods Analysis Plan Other*
**Data sharing**		
**Definitions:** ‘Data’ refers to recorded information that supports the analyses reported in the article. A ‘data availability statement’ can be as simple as a url link to a data file, or as complex as a written explanation as to why data cannot be shared.		
**Coder instructions:** Check the article for a data availability statement/link. They are often located in the ‘supplementary material’, ‘acknowledgements’, ‘author notes’, ‘methods’ or ‘results’ sections. Search the article for the text ‘data available’ (to cover ‘data availability’ and ‘data available’).		
Data availability statement	Does the article state whether or not data are available?	Yes – the statement says that the data (or some of the data) are available / Yes – the statement says that the data are NOT available / No – there is no data availability statement / Other*
Data sharing method	How does the statement indicate the data are available?	On request from the authors / personal or institution website / An online, third-party repository (eg, OSF, FigShare) / supplementary materials hosted by the journal / Other*
Data accessibility	Can you access, download, and open the data files?	Yes / No / Other*
Data documentation	Are the data files clearly documented?	Yes / No / Other*
Data content	Do the data files appear to contain all of the raw data necessary to reproduce the reported findings?	Yes / No / Unclear / Other*
**Materials sharing**		
**Definitions:** ‘Materials’ refers to any study items that would be needed to repeat the study, such as stimuli, survey instruments and computer code/software used for data collection, presentation stimuli or running experiments.		
Materials availability statement	Does the article state whether or not materials are available?	Yes – the statement says that the materials (or some of the materials) are available / Yes – the statement says that the materials are NOT available / No – there is no materials availability statement / Other*
Materials sharing method	According to the statement, how are the materials accessible?	On request from the authors / personal or institution website / An online, third-party repository (eg, OSF, FigShare) / supplementary materials hosted by the journal / Other*
Materials accessibility	Can you access, download and open the materials files?	Yes / No / Other*
**Analysis script sharing**		
**Definition:** ‘Analysis scripts’ refers to specification of data preparation and analysis steps in the form of highly detail step-by-step instructions for using point-and-click software, analysis code (eg, R), or syntax (eg, from SPSS).		
**Coder instructions:** Check the article for an analysis script availability statement/link. They are often located in the ‘supplementary material’, ‘acknowledgements’, ‘author notes’, ‘methods’ or ‘results’ sections. Search for the text ‘analysis script’ and ‘analysis code’.		
Analysis script availability statement	Does the article state whether or not analysis scripts are available?	Yes – the statement says that the analysis scripts (or some of the analysis scripts) are available / Yes – the statement says that the analysis scripts are NOT available / No – there is no analysis script availability statement
Analysis script sharing method	According to the statement, how are the analysis scripts accessible?	On request from the authors / Personal or institution website / An online, third-party repository (eg, OSF, FigShare) / supplementary materials hosted by the journal / Other*
Analysis script accessibility	Can you access, download, and open the analysis script files?	Yes / No / Other*
**Funding**		
**Coder instructions**: Funding is usually reported in a specific section, for example, ‘Author information’, or ‘Funding statement’. Search the article for the phrase ‘funding’. If you are unsure whether an organisation is an activity-related company, pharmaceutical company, other private company or public organisation, Google the organisation name and code accordingly. If it is unclear to you whether the funding is private or public, choose the ‘other’ option and enter ‘unclear’.		
Funding statement	Does the article include a statement indicating whether there were funding sources?	Yes – the statement says that there was funding from an activity-related company (eg, Nike, Fitbit) / Yes – funding from a pharmaceutical company (eg, Pfizer, GSK)/ Yes – funding from another private company (eg, Google, Coca Cola) / Yes – funding from a public organisation (eg, National Institute of Health Research)/ Yes – the statement says that there was no funding was provided / No – there is no funding statement / Unclear / Other*
**Conflict of interest**		
**Coder instructions:** Conflicts of interest are usually reported in a specific section, for example, ‘Author information’ or ‘Conflict of interest statement’. Search the article for the phrases ‘conflict of interest’ and/or ‘competing interest’. If you are unsure whether an organisation is an activity-related company, pharmaceutical company, other private company or public organisation, Google the organisation name and code accordingly. If it is unclear to you whether the conflict of interest is private or public, choose the ‘other’ option and enter ‘unclear’.		
Conflict of interest statement	Does the article include a statement indicating whether there were any conflicts of interest?	Yes – the statement says that there was a conflict of interest from an activity-related company (eg, Nike, Fitbit) / Yes – conflict of interest from a pharmaceutical company (eg, Pfizer, GSK)/ / Yes – conflict of interest from another private company (eg, Google, Coca Cola) / Yes – conflict of interest from a public organisation (eg, National Institute of Health Research)/ Yes – the statement says that there is no conflict of interest / No – there is no conflict of interest statement / Other*
**Replication**		
**Definitions:** ‘Replication’ refers to repetition of a previous study’s methods in order to ascertain whether similar findings can be obtained.		
**Coder instructions**: Search the abstract and introduction for the phrase ‘replicat’ (to cover ‘replication’, ‘replicates’ etc). Confirm the authors are using the phrase with the definition provided above.		
Replication statement	Does the article claim to report a replication study?	The article claims to report a replication study (or studies) / There is no clear statement that the article reports a replication study (or studies) / Other*
**Open access**		
**Coder instructions:** To establish the open access status of the article: Go to https://openaccessbutton.org/ and enter the article’s doi (eg, ‘10.1371/journal.pcbi.1004574’) if available (if not, enter the article title). If a link is provided, check that you can access the article at the link. If the article is accessible answer ‘Yes’. If the article is not accessible at the provided link, or no link is provided, answer ‘No’.		
Open access status	Is the article open access?	Yes – found via open access button / Yes – found via other means / No – could not access article other than through paywall / Other

*If a response marked with an asterisk is selected, the coder is asked to provide more detail in a free-text response box.

### Procedure

Coding of identified intervention reports took place between July and September 2021, with all data extracted onto a Google Form.^35^ All reports were independently coded by two researchers (IS coded all 100 papers, EN and OC coded 50 each). Any discrepancies were resolved through discussion, with input from a third researcher who was not involved in the initial coding of that specific paper (EN or OC).

### Analysis

Raw numbers and percentages were identified for each variable. Inter-rater reliability of the independent coding by the two researchers, prior to any changes after discrepancy discussions, was calculated using Krippendorff’s alpha^36^ using R package ‘irr’ V.0.84.1,^37^ as performed in other related research coding studies.^22 38^

## Results

### Sample characteristics

Twenty-two out of the 100 physical activity behaviour change intervention reports were published in 2018, 33 in 2019, 37 in 2020 and 8 in 2021. The 100 reports evaluated studies conducted in 24 different countries, taking place most commonly in the USA (n=24), Australia (n=19), Canada (n=10) and the UK (n=7). A full summary of countries in included reports is presented in online supplemental file 2.

10.1136/bmjsem-2021-001282.supp2 Supplementary data

### Open Science practices in physical activity behaviour change intervention reports

Final reconciled coding of Open Science practices for all 100 included physical activity behaviour change intervention reports can be found in online supplemental file 3.

10.1136/bmjsem-2021-001282.supp3 Supplementary data

### Article availability (open access)

**Figure 2:**
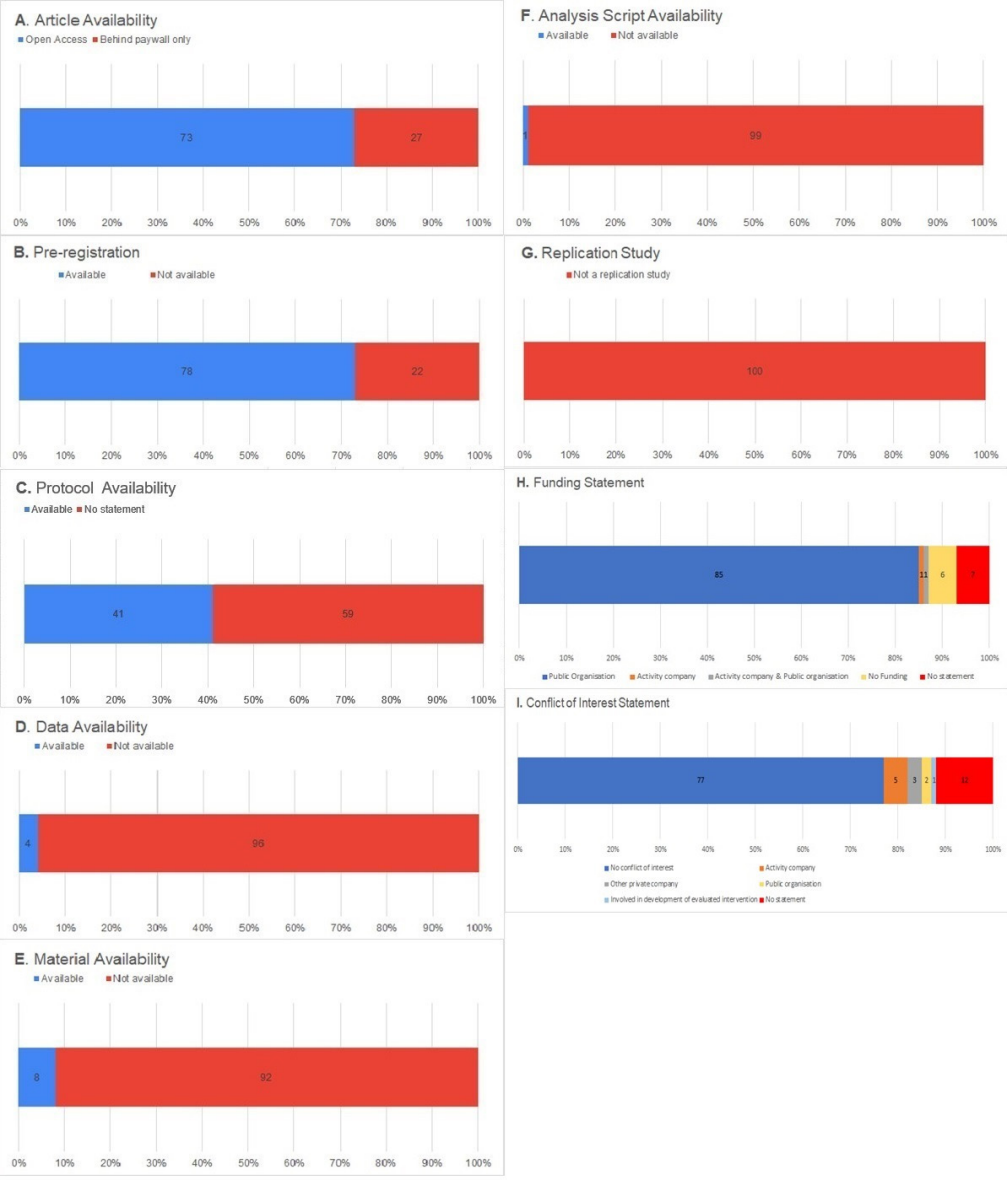
(A) Article availability, (B) pre-registration, (C) protocol availability, (D) data availability, (E) material availability, (F) analysis script availability, (G) replication study, (H) funding statement and (I) conflict of interest statement.

Seventy-three out of 100 physical activity behaviour change intervention reports were available via open access, with 27 of them only accessible behind a paywall (figure 2A).

### Pre-registration

Seventy-eight out of 100 physical activity behaviour change intervention reports included a statement indicating existence of a study pre-registration. Of those, 77 could be accessed. Forty-three of all accessible pre-registrations were recorded prospectively (ie, before data collection commenced) and 34 were recorded respectively (ie, after data collection commenced). Seventy-seven of all accessible pre-registrations declared specifications relating to study methods, 24 declared hypotheses and 5 declared analysis plans. Thirty-seven of all accessible pre-registrations were hosted on ClinicalTrials.gov (48.1%), 18 on the Australian and New Zealand Clinical Trials Registry (ANZCTR: 23.4%), 14 on the International Standard Randomised Clinical Trial Number registry (ISRCTN: 18.2%), 3 on Netherlands Trial Register (NTR: 3.9%) and 1 on Deutsches Register Klinischer Studien (DRKS), Iranian Registry of Clinical Trials (IRCT), Registro Brasileiro de Ensaios Clinicos (REBEC), UMIN Clinical Trials Registry (UMIN-CTR) and Chinese Clinical Trial Registry (ChCTR) (1.3% each). One included study was a Registered Report,^39^ logged with an International Registered Report Identifier (1.3%) (figure 2B).

### Protocol sharing

Forty-one out of 100 physical activity behaviour change intervention reports included a statement about protocol availability. All 41 (100%) of these protocols specified study methods, 40 (97.6%) specified analysis plans and 14 (34.1%) specified hypotheses (figure 2C).

### Data sharing

Thirty-two out of 100 physical activity behaviour change intervention reports included a data availability statement. Of those, 22 stated data were only available on request from the authors, 5 stated that data were available within the reports’ supplementary files, 1 stated that data were available via a personal or institutional website and 4 stated that data were not available. Only 4 out of these 32 reports included a data availability statement that data files that were actually accessible to download, with two of these providing clear documentation for the data files and two providing sufficient detail needed to reproduce findings (figure 2D).

### Material sharing

Seventeen out of 100 physical activity behaviour change intervention reports included a materials availability statement. Of those, 10 reports stated that materials were available within the reports’ supplementary files and 7 stated that materials were only available on request from the authors. Eight out of the 10 studies which stated that materials were provided as supplementary files actually provided accessible and downloadable materials, such as full or sample intervention activities (figure 2E).

### Analysis script sharing

One out of 100 physical activity behaviour change intervention reports included an analysis script availability statement,^40^ with this provided as a supplementary file (figure 2F).

### Replication study

None of the 100 physical activity behaviour change intervention reports were described as replication studies (figure 2G).

### Funding

Ninety-three out of the 100 physical activity behaviour change intervention reports included a statement about funding sources. Most of the reports disclosed public funding only, such as via government-funded research grants, charities or universities (n=85). One report disclosed both public funding and funding from private activity-related companies^41^ and one report disclosed funding from private activity-related companies only.^42^ Six reports reported receiving no funding (figure 2H).

### Conflicts of interest

Eighty-eight out of the 100 articles provided a conflict of interest statement. Most of these reports stated that there were no conflicts of interest (n=77). Eleven reports stated that there was at least one conflict of interest, including from an activity company (n=5), a public organisation such as government or charities (n=2), a pharmaceutical company (n=1), a non-activity or pharmaceutical company (n=1), a combination of activity, pharmaceutical and other private companies (n=1), or that researchers were involved in the development and evaluation of the reported intervention (n=1) (figure 2I).^43^

### Inter-rater reliability assessment

Inter-rater reliability of all coding across the 100 reports was assessed as good, *a*=0.73.

## Discussion

This study aimed to assess Open Science practices within physical activity behaviour change intervention reports. It was found that Open Science practices varied among the assessed 100 physical activity behaviour change intervention reports. Most reports were open access and pre-registered, with reported funding sources and conflicts of interest. However, open materials, data and analysis scripts were not frequently provided and no replication studies were identified.

Pre-registration of studies was found to be slightly more common for physical activity intervention RCTs (78%), than found in smoking cessation intervention RCTs (73%)^22^ and much more common than in wider psychological research of varying study designs (3%).^21^ In our study, similar amounts of studies were pre-registered prospectively (55.7%: prior to data collection commencing) or retrospectively (44.2%: after data collection had commenced),^30^ although this distinction between pre-registrations has not been assessed in comparable research. The common prevalence of retrospective pre-registration via clinical trials is arguably not true pre-registration, nor transparent from the study’s outset.^12 13^ One included study was noted as a Registered Report,^39^ where in-principle acceptance to journals is given based on study proposals at conception stage, rather than based on completed studies and their reported findings.^44 45^ No Registered Reports were identified in smoking cessation^22^ and psychology,^21^ perhaps reflecting a slow increase in Registered Report numbers over time.^15^ Protocols were available as separate papers or linked publications in 41% of included physical activity studies, which is higher than in smoking cessation studies (29%)^22^; and wider psychology research (0%).^21^ The increased prevalence of protocols within physical activity and smoking cessation likely reflects greater availability of health-related protocol publications,^46^ via specific journals such as JMIR Research Protocols and via protocols as specific types of publications within wider journals such as BMC Public Health and Trials. High prevalence of protocols in this study is also indicative of RCTs being both a common study design in health and intervention research^47^ and a study design typically accompanied by research protocols.^48^

Open access reports were at similarly moderate levels in physical activity (73%) than in smoking cessation (71%)^22^; and psychology (65%),^21^ but greater than the 45% observed in the social sciences,^20^ the 45% across scientific literature published in 2015^16^ and the 25% in biomedicine.^23^ This high rate of open access publishing in physical activity interventions may reflect increasing requirements by health funding bodies for open access publications,^49^ as well as increasing usage of preprint servers such as medRxiv for medical sciences and PsyArXiv for the psychological sciences.^50^

Open materials were less commonly available in physical activity reports (8%) than in smoking cessation reports (13%),^22^ psychology (14%)^21^; and biomedicine (33%).^23^ Open data were also less common across physical activity reports (4%) than in smoking cessation reports (7%),^22^ but greater than the 2% of wider psychological research.^21^ Provision of raw data as supplementary files to published intervention reports or via trusted third-party repositories such as the Open Science Framework is important to facilitate evidence synthesis. Open analysis scripts were found to be as infrequently provided in physical activity studies than in smoking interventions and wider psychological research (all 1%).^21 22^ No replication attempts were identified in this sample of physical activity intervention reports, same as within smoking cessation reports^22^ but less than in the social sciences (1%)^20^; and in wider psychology studies (5%).^21^

Declaration of funding sources were declared in physical activity reports (93%) similarly to smoking cessation reports (95%)^22^; more so than wider psychology (62%),^21^ social sciences (31%)^20^ and biomedical science reports (69%).^23^ Similarly, a conflict of interest statement was provided as commonly in physical activity reports than in smoking cessation reports (88% in both)^22^ and higher than in wider psychology (39%),^21^ social sciences (39%)^20^ and biomedical sciences reports (65%).^23^ Eight per cent of studies reported conflicts from private companies including activity, pharmaceutical and other companies, less than the 20% of studies reporting company funding in smoking cessation interventions.^22^

### Future steps to increase Open Science in physical activity interventions

This research has demonstrated a need to address the low levels of Open Science engagement in physical activity research, particularly in the areas of open materials, data, analysis scripts and replication attempts. As with any complex behaviour change, this transformation requires systems change across bodies involved in the development, running and publication of physical activity research: researchers, research institutions, funding organisations, journals and beyond.^1 9^ In order to develop effective behaviour change interventions, it is important to use a systematic and comprehensive approach to intervention development, underpinned by a model of behaviour and theoretically predicted mechanisms of action.^51–53^ The Capability, Opportunity, Motivation, Behaviour model^54^ posits that changing behaviour involves changing one or more of the following: capability (psychological and physical capacity to engage in the behaviour), opportunity (external factors that make the execution of a particular behaviour possible or prompt it) and motivation (internal processes that energise and direct behaviour). We argue that understanding the capability, opportunity and motivation associated with Open Science practices^9^ and developing interventions to address these determinants of behaviour change,^55^ is key to increase engagement with Open Science.

For example, low perceived capability towards Open Science practices in physical activity researchers can be addressed by providing researchers with training tailored to the context of activity intervention research (eg, online training on how to make anonymised activity monitor data openly available, how to use preprint servers most relevant to activity research or how to make their activity analysis reproducible). Opportunity to engage in Open Science practices can be facilitated within institutions, encouraging discussions around Open Science in the context of physical activity research^19^ and in science more broadly,^21 23 56^ as well as developing a research culture valuing and promoting the benefits of Open Science practices.^16 23^ Motivation for Open Science can be addressed by providing incentives, such as awarding funding to research-embedding open practices.^57^ Similarly, Open Science badges recognising open data, materials and pre-registration have been adopted by journals as a simple, low-cost scheme to reward these research behaviours.^58^ However, uptake of Open Science badges in physical activity journals is currently low and is rife for increased uptake in the field.

### Strengths and limitations

A strength of this study is the implementation of a comprehensive and previously used approach to identify Open Science practices. Moreover, two researchers independently carried out the coding of Open Science practices, reducing the risk of human error and maximising reliability.^59^ A limitation is that the search and screening processes were conducted by a single author. However, unlike systematic reviews, we did not attempt to conduct a comprehensive search to identify all relevant research but to select a somewhat random subsample to analyse Open Science practices and inform specific recommendations for future research. In this regard, it is worth acknowledging that results are based on a relatively small sample of physical activity behaviour change reports, meaning findings may not be applicable to all physical activity research. Last, the assessment of Open Science practices was entirely dependent on what was described within evaluation reports. Direct requests to authors or additional wider searching of third-party registries such as Open Science Framework may have identified additional information.

## Conclusions

Open Science practices in physical activity behaviour change intervention reports were varied. Open access publication and pre-registration of research plans were common, although pre-registration was often done retrospectively, that is, after data collection has started, hence not in the most transparent manner. Provision of open data, materials and analysis was rare and replication attempts were non-existent. Funding sources and conflicts of interest were usually declared. Urgent initiatives are needed to increase the uptake of all Open Science practices in physical activity, with a particular focus on open materials, data, analysis scripts and replication attempts.

## Data Availability

Data are available in a public, open access repository. All data from this study are available here: https://osf.io/t5gw4/
