# Supplementary material for: Assessing Open Science practices in physical activity behaviour change intervention evaluations
Source: BMJ Open Sport Exerc Med. 2022 May 23;8(2):e001282. doi: 10.1136/bmjsem-2021-001282 (PMC9174779; doi:10.1136/bmjsem-2021-001282)
Supplement: Supplementary data [file bmjsem-2021-001282supp001.pdf]

Supplementary File 1. Updates to pre-registration

During the course of this study and peer review, we made minor adjustments to the pre-registered protocol:

1. We added an additional item: 'Was the declared pre-registration actually pre-registered ahead of data collection?' (Question 8). This specified whether a declared study pre-registration was registered ahead of data collection, or whether it was actually retrospectively registered after data collection had commenced. Responses for this new item were 'Yes – this pre-registration was registered ahead of data collection' or 'No – this pre-registration was retrospectively registered after data collection had commenced'
2. The presence of 'supplementary material' was not sufficient for Material availability alone, as supplementary materials feature a variety of documents from protocols, materials, data etc.
3. Where a data and/or materials availability sub-heading was present in a paper, but no discussion of data and/or materials availability was actually given there; we coded the paper as 'No - there is no data availability statement'/'No - there is no materials availability statement'.
4. We added an option 'Yes - Funded by a non-profit' under Funding sources.
5. We added an option 'Yes - Funded by a non-profit' under Conflict of Interest.
6. We added an option 'Yes - Statement says researchers involved in both development and evaluation of intervention' under Conflict of Interest.
