# Supplementary material for: Assessing Open Science practices in physical activity behaviour change intervention evaluations
Source: BMJ Open Sport Exerc Med. 2022 May 23;8(2):e001282. doi: 10.1136/bmjsem-2021-001282 (PMC9174779; doi:10.1136/bmjsem-2021-001282)
Supplement: Supplementary data [file bmjsem-2021-001282supp002.pdf]

Supplementary File 2. Country of origin of included articles.

**Country**	**n articles**
Australia	19
Austria	1
Belgium	4
Brazil	2
Canada	10
China	1
Colombia	1
Costa Rica	1
Germany	3
Hong Kong	1
Iran	1
Ireland	1
Italy	1
Japan	1
Netherlands	5
Norway	4
Singapore	1
South Korea	1
Spain	4
Sweden	4
Switzerland	1
Taiwan	2
UK	7
USA	24
**Total**	100
