# Supplementary material for: Assessing Open Science practices in physical activity behaviour change intervention evaluations
Source: BMJ Open Sport Exerc Med. 2022 May 23;8(2):e001282. doi: 10.1136/bmjsem-2021-001282 (PMC9174779; doi:10.1136/bmjsem-2021-001282)
Supplement: Supplementary data [file bmjsem-2021-001282supp003.pdf]

Supplementary File 3. Open Science markers assessed in 100 physical activity behaviour change intervention evaluation reports.

Study	Pre-registration available	Protocol available	Data available	Materials available	Analysis script available	Replication study	Open Access	Funding statement	Conflict of interest statement
Alhassan et al. 2019 [1]	No	No	No	No	No	No	No	No funding	No
Alley et al. 2018 [2]	Yes	Yes	No - Upon request only	No	No	No	Yes	Yes – Public organisation	No conflict of interest
Andersen et al. 2020 [3]	Yes	No	No - Upon request only		No	No	Yes	Yes – Public organisation	No conflict of interest
Balducci et al. 2019 [4]	Yes	Yes	No - Upon request only	Yes – Supplementary files	No	No	Yes	Yes – Public organisation	Yes – Activity company & pharmaceutical company & other private company
Barrett et al. 2018 [5]	Yes	No	No - Upon request only	No	No	No	Yes	No	No conflict of interest
Belton et al. 2019 [6]	Yes	No	No - Upon request only	No	No	No	Yes	Yes – Public organisation	No conflict of interest
Berendsen et al. 2020 [7]	Yes	Yes	No	No	No	No	Yes	Yes – Public organisation	No conflict of interest
Berglind et al. 2020 [8]	Yes	Yes	No	No	No	No	Yes	Yes – Public organisation	No conflict of interest
Bock et al. 2019 [9]	Yes	Yes	No	No	No	No	Yes	Yes – Public organisation	No conflict of interest
Boudreau et al. 2020 [10]	Yes	Yes	No	No	No	No	Yes	Yes – Public organisation	No conflict of interest
Catenacci et al. 2019 [11]	Yes	No	No - Upon request only	No	No	No	Yes	Yes – Public organisation	Yes – Activity company
Chiang et al. 2020 [12]	No	No	No	No	No	No	No	Yes – Public organisation	No conflict of interest
Chow et al. 2021 [13]	Yes	No	No	Yes – Supplementary files	No	No	No	Yes – Public organisation	No conflict of interest
Coolkens et al. 2018 [14]	No	No	No	No	No	No	Yes	No funding	No

Corder et al. 2020 [15]	Yes	Yes	No - Upon request only	No	No	No	Yes	Yes – Public organisation	No conflict of interest
Costigan et al. 2018 [16]	Yes	No	No	No	No	No	No	Yes – Public organisation	No conflict of interest
Dennett et al. 2018 [17]	Yes	No	No	No	No	No	Yes	Yes – Public organisation	No conflict of interest
Driediger et al. 2019 [18]	No	No	No	No	No	No	Yes	Yes – Public organisation	No conflict of interest
Edney et al. 2020 [19]	Yes	Yes	No - Upon request only	No – Supplementary files	No	No	No	Yes – Public organisation	No conflict of interest
Ek et al. 2020 [20]	Yes	Yes	No	No	No	No	Yes	Yes – Public organisation	No conflict of interest
Fischer et al. 2019 [21]	Yes	Yes	No	No	No	No	Yes	Yes – Public organisation	No conflict of interest
Freitas et al. 2021 [22]	Yes	No	No	No	No	No	No	Yes – Public organisation	No conflict of interest
Fukuoka et al. 2019 [23]	Yes	Yes	No	No	No	No	Yes	Yes – Public organisation	Yes – Public organisation
Galarraga et al. 2020 [24]	Yes	Yes	No	No	No	No	Yes	Yes – Public organisation	No
Garcia-Ortiz et al. 2018 [25]	Yes	Yes	No	No	No	No	Yes	Yes – Public organisation	No conflict of interest
Golsteijn et al. 2018 [26]	Yes	No	No - Upon request only	No - Upon request only	No	No	Yes	Yes – Public organisation	Yes – Other private company
Guagliano et al. 2020 [27]	Yes	Yes	No - Upon request only	No - Upon request only	No	No	Yes	Yes – Public organisation	No conflict of interest
Gutierrez-Martinez et al. 2018 [28]	No	No	No	No - Upon request only	No	No	Yes	Yes – Public organisation	No conflict of interest
Ha et al. 2020 [29]	Yes	Yes	No	No	No	No	Yes	Yes – Public organisation	No conflict of interest
Hamilton et al. 2020 [30]	No	No	No	No	No	No	No	Yes – Public organisation	No

Hardeman et al. 2020 [31]	Yes	Yes	No - Upon request only	No	No	No	Yes	Yes – Public organisation	No conflict of interest
Harrington et al. 2018 [32]	Yes	Yes	No	No	No	No	Yes	Yes – Public organisation	No conflict of interest
Isensee et al. 2018 [33]	Yes	Yes	No	No	No	No	No	Yes – Public organisation	No
Jago et al. 2019 [34]	Yes	Yes	Yes - Supplement ary files	No	No	No	Yes	Yes – Public organisation	No conflict of interest
Jung et al. 2020 [35]	Yes	Yes	No - Upon request only	No	No	No	Yes	Yes – Public organisation	Yes – Activity company
Kayser et al. 2019 [36]	No	Yes	No	No	No	No	No	Yes – Activity company & public organisation	Yes – Activity company
Keller et al. 2020 [37]	Yes	No	Yes – Supplement ary files	Yes – Supplemen tary files	No	No	Yes	Yes – Public organisation	No conflict of interest
Kernot et al. 2019 [38]	Yes	Yes	No	No	No	No	No	Yes – Public organisation	No
Kerr et al. 2018 [39]	Yes	Yes	No - Upon request only	No	No	No	Yes	Yes – Public organisation	No conflict of interest
Kolle et al. 2020 [40]	Yes	No	No - Upon request only	No	No	No	Yes	Yes – Public organisation	No conflict of interest
Lo et al. 2021 [41]	Yes	No	No	No	No	No	Yes	No funding	No conflict of interest
Looyestyn et al. 2018 [42]	Yes	No	No	No	No	No	Yes	No	No conflict of interest
Lugones-Sanchez et al. 2020 [43]	Yes	Yes	No	No	No	No	Yes	Yes – Public organisation	No conflict of interest
Lundqvist et al. 2020 [44]	Yes	No	No - Upon request only	No	No	No	Yes	Yes – Public organisation	No conflict of interest
Lynch et al. 2019 [45]	Yes	Yes	No	No	No	No	Yes	Yes – Public organisation	No conflict of interest
Ma et al. 2019 [46]	Yes	No	No - Upon request only	No	No	No	No	Yes – Public organisation	No conflict of interest

Mama et al. 2020 [47]	No	No	No	No	No	No	Yes	Yes – Public organisation	No conflict of interest
Mascarenhas et al. 2018 [48]	Yes	No	No	No	No	No	Yes	No	No conflict of interest
Maxwell-Smith et al. 2019 [49]	Yes	Yes	No	No	No	No	Yes	Yes – Public organisation	No conflict of interest
McNeil et al. 2018 [50]	Yes	No	No - Upon request only	No - Upon request only	No	No	Yes	Yes – Public organisation	No conflict of interest
McNeil et al. 2019 [51]	Yes	No	No	No	No	No	Yes	Yes – Public organisation	No conflict of interest
Meng et al. 2018 [52]	No	No	No	No	No	No	Yes	Yes – Public organisation	No conflict of interest
Mitchell et al. 2019 [53]	No	Yes	No	No	No	No	No	Yes – Public organisation	No
Morris et al. 2019 [54]	No	No	No	No	No	No	No	No	No
Muellmann et al. 2019 [55]	Yes	No	No	No	No	No	Yes	Yes – Public organisation	No conflict of interest
Muller-Riemenschneider et al. 2020 [56]	Yes	Yes	No - Upon request only	No - Upon request only	No	No	Yes	Yes – Public organisation	No conflict of interest
Murawski et al. 2019 [57]	Yes	Yes	No	No	No	No	No	Yes – Public organisation	No conflict of interest
Nathan et al. 2020 [58]	Yes	No	No	No	No	No	Yes	Yes – Public organisation	No conflict of interest
Nooijen et al. 2020 [59]	Yes	Yes	No - Upon request only	No	No	No	Yes	Yes – Activity company	Yes – Activity company
Nourizadeh et al. 2020 [60]	Yes	No	No	No	No	No	No	No	No conflict of interest
Ojeda-Rodriguez et al. 2021 [61]	No	No	No	No	No	No	No	Yes – Public organisation	No conflict of interest
Ozemek et al. 2020 [62]	No	No	No	No	No	No	No	Yes – Public organisation	No conflict of interest
Pallante et al. 2019 [63]	No	No	No	No	No	No	Yes	No	No

Park et al. 2020 [64]	No	No	No	No	No	No	No	Yes – Public organisation	No conflict of interest
Patten et al. 2019 [65]	No	No	No	No	No	No	No	Yes – Public organisation	No conflict of interest
Plow et al. 2019 [66]	Yes	No	No	No	No	No	No	Yes – Public organisation	Yes – Pharmaceutical company
Pope et al. 2018 [67]	No	No	No	Yes – Supplementary files	No	No	Yes	Yes – Public organisation	No conflict of interest
Pope et al. 2019 [68]	Yes	No	No	No	No	No	Yes	Yes – Public organisation	No conflict of interest
Poppe et al. 2019 [69]	Yes	Yes	No	Yes – Supplementary files	No	No	Yes	Yes – Public organisation	Yes – researchers involved in development & evaluation of intervention
Prado et al. 2020 [70]	Yes	No	No	No	No	No	No	Yes – Public organisation	No conflict of interest
Reed et al. 2018 [71]	No	No	No	Yes – Supplementary files	No	No	Yes	Yes – Public organisation	No conflict of interest
Reedman et al. 2019 [72]	Yes	Yes	No	No	No	No	Yes	Yes – Public organisation	Yes – Activity company
Reich et al. 2020 [73]	Yes	No	No	No	No	No	Yes	Yes – Public organisation	No conflict of interest
Reid et al. 2021 [74]	Yes	No	No	No	No	No	Yes	Yes – Public organisation	No conflict of interest
Riiser et al. 2020 [75]	Yes	Yes	No	No	No	No	Yes	Yes – Public organisation	No conflict of interest
Robbins et al. 2019 [76]	Yes	Yes	No	No	No	No	Yes	Yes – Public organisation	No conflict of interest
Robinson et al. 2019 [77]	Yes	No	No	Yes – Supplementary files	No	No	Yes	Yes – Public organisation	No conflict of interest

Rodriguez-Hernandez et al. 2019 [78]	No	No	Yes – Supplementary files	No	No	No	Yes	No funding	No conflict of interest
Rollo et al. 2020 [79]	Yes	No	No	No	No	No	Yes	Yes – Public organisation	No conflict of interest
Ruissen et al. 2018 [80]	Yes	No	No	No - Upon request only	No	No	No	Yes – Public organisation	No
Sala et al. 2021 [81]	Yes	No	No	No	No	No	Yes	Yes – Public organisation	No conflict of interest
Sanchez-Lopez et al. 2020 [82]	Yes	No	No	No	No	No	Yes	No funding	No conflict of interest
Santos et al. 2019 [83]	Yes	No	No	No	No	No	No	No	No conflict of interest
Sebire et al. 2018 [84]	Yes	Yes	No - Personal or institution website	No	No	No	Yes	Yes – Public organisation	No conflict of interest
Seljebotn et al. 2019 [85]	No	No	No	No	No	No	Yes	Yes – Public organisation	No conflict of interest
Singh et al. 2020 [86]	Yes	No	No - Supplementary files	No – Supplementary files	No	No	No	Yes – Public organisation	No
Spring et al. 2018 [87]	Yes	Yes	No	No	No	No	Yes	Yes – Public organisation	No conflict of interest
Taylor et al. 2020 [88]	Yes	Yes	No - Upon request only	No	No	No	Yes	Yes – Public organisation	Yes – Public organisation
Tudor-Locke et al. 2020 [89]	Yes	Yes	Yes – Supplementary files	No	Yes – Supplementary files	No	No	Yes – Public organisation	No
Uemura et al. 2021 [90]	Yes	No	No	No	No	No	No	Yes – Public organisation	No conflict of interest
van den Berg et al. 2019 [91]	No	No	No	No	No	No	Yes	Yes – Public organisation	No conflict of interest

van Dyck et al. 2019 [92]	Yes	No	No	Yes – Supplementary files	No	No	Yes	Yes – Public organisation	No conflict of interest
van Hoye et al. 2018 [93]	Yes	Yes	No - Upon request only	No - Upon request only	No	No	Yes	Yes – Public organisation	No conflict of interest
Vandelanotte et al. 2021 [94]	Yes	Yes	No - Upon request only	No	No	No	No	Yes – Public organisation	No conflict of interest
Veldman et al. 2020 [95]	Yes	No	No	No	No	No	Yes	Yes – Public organisation	No conflict of interest
Viester et al. 2018 [96]	Yes	No	No	No	No	No	Yes	Yes – Public organisation	No conflict of interest
Volders et al. 2020 [97]	Yes	Yes	No	No	No	No	Yes	Yes – Public organisation	No conflict of interest
Wadsworth et al. 2020 [98]	No	No	No	No	No	No	Yes	No funding	No conflict of interest
Xu et al. 2020 [99]	Yes	No	No	No	No	No	Yes	Yes – Public organisation	No conflict of interest
Yoong et al. 2019 [100]	Yes	No	No	No	No	No	Yes	Yes – Public organisation	No conflict of interest
**Total**	**78/100**	**41/100**	**4/100**	**8/100**	**1/100**	**0/100**	**73/100**	**93/100 reported**	**88/100 reported**

References

[1] S. Alhassan, C. W. St Laurent, S. Burkart, C. J. Greever, and M. N. Ahmadi, "Feasibility of Integrating Physical Activity Into Early Education Learning Standards on Preschooler's Physical Activity Levels," *J. Phys. Act. Health*, vol. 16, no. 2, pp. 101–107, Feb. 2019, doi: 10.1123/jpah.2017-0628.

[2] S. J. Alley *et al.*, "The effectiveness of a web 2.0 physical activity intervention in older adults – a randomised controlled trial," *Int. J. Behav. Nutr. Phys. Act.*, vol. 15, no. 1, p. 4, Dec. 2018, doi: 10.1186/s12966-017-0641-5.

[3] E. Andersen, S. Øvreås, K. A. Jørgensen, J. Borch-Jenssen, and T. Moser, "Children's physical activity level and sedentary behaviour in Norwegian early childhood education and care: effects of a staff-led cluster-randomised controlled trial," *BMC Public Health*, vol. 20, no. 1, p. 1651, Nov. 2020, doi: 10.1186/s12889-020-09725-y.

[4] S. Balducci *et al.*, "Effect of a Behavioral Intervention Strategy on Sustained Change in Physical Activity and Sedentary Behavior in Patients With Type 2 Diabetes: The IDES_2 Randomized Clinical Trial," *JAMA*, vol. 321, no. 9, pp. 880–890, Mar. 2019, doi: 10.1001/jama.2019.0922.

[5] S. Barrett, S. Begg, P. O'Halloran, and M. Kingsley, "Integrated motivational interviewing and cognitive behaviour therapy can increase physical activity and improve health of adult ambulatory care patients in a regional hospital: the Healthy4U randomised controlled trial," *BMC Public Health*, vol. 18, p. 1166, Oct. 2018, doi: 10.1186/s12889-018-6064-7.

[6] S. Belton, A. McCarren, B. McGrane, D. Powell, and J. Issartel, "The Youth-Physical Activity Towards Health (Y-PATH) intervention: Results of a 24 month cluster randomised controlled trial," *PLOS ONE*, vol. 14, no. 9, p. e0221684, Sep. 2019, doi: 10.1371/journal.pone.0221684.

[7] B. A. J. Berendsen, M. R. C. Hendriks, G. M. Rutten, S. P. J. Kremers, H. H. C. M. Savelberg, and N. C. Schaper, "The added value of frequent physical activity group sessions in a combined lifestyle intervention: A cluster randomised trial in primary care," *Prev. Med. Rep.*, vol. 20, p. 101204, Dec. 2020, doi: 10.1016/j.pmedr.2020.101204.

[8] D. Berglind, D. Yacaman-Mendez, C. Lavebratt, and Y. Forsell, "The Effect of Smartphone Apps Versus Supervised Exercise on Physical Activity, Cardiorespiratory Fitness, and Body Composition Among Individuals With Mild-to-Moderate Mobility Disability: Randomized Controlled Trial," *JMIR MHealth UHealth*, vol. 8, no. 2, p. e14615, Feb. 2020, doi: 10.2196/14615.

[9] B. C. Bock *et al.*, "Exercise Videogames, Physical Activity, and Health: Wii Heart Fitness: A Randomized Clinical Trial," *Am. J. Prev. Med.*, vol. 56, no. 4, pp. 501–511, Apr. 2019, doi: 10.1016/j.amepre.2018.11.026.

[10] F. Boudreau *et al.*, "Effectiveness of a web-based computer-tailored intervention promoting physical activity for adults from Quebec City: a randomized controlled trial," *Health Psychol. Behav. Med.*, vol. 8, no. 1, pp. 601–622, Jan. 2020, doi: 10.1080/21642850.2020.1850287.

[11] V. A. Catenacci *et al.*, "The Impact of Timing of Exercise Initiation on Weight Loss: An 18-Month Randomized Clinical Trial," *Obes. Silver Spring Md*, vol. 27, no. 11, pp. 1828–1838, Nov. 2019, doi: 10.1002/oby.22624.

[12] S.-L. Chiang, C.-L. Shen, L.-C. Chen, Y.-P. Lo, C.-H. Lin, and C.-H. Lin, "Effectiveness of a Home-Based Telehealth Exercise Training Program for Patients With Cardiometabolic Multimorbidity: A Randomized Controlled Trial," *J. Cardiovasc. Nurs.*, vol. 35, no. 5, pp. 491–501, Oct. 2020, doi: 10.1097/JCN.0000000000000693.

[13] E. J. Chow *et al.*, "Feasibility of a behavioral intervention using mobile health applications to reduce cardiovascular risk factors in cancer survivors: a pilot randomized controlled trial," *J. Cancer Surviv.*, vol. 15, no. 4, pp. 554–563, Aug. 2021, doi: 10.1007/s11764-020-00949-w.

[14] R. Coolkens, P. Ward, J. Seghers, and P. Iserbyt, "The Effect of Organized Versus Supervised Recess on Elementary School Children's Participation, Physical Activity, Play, and Social Behavior: A Cluster Randomized Controlled Trial," *J. Phys. Act. Health*, vol. 15, no. 10, pp. 747–754, Oct. 2018, doi: 10.1123/jpah.2017-0591.

[15] K. Corder *et al.*, "Effectiveness and cost-effectiveness of the GoActive intervention to increase physical activity among UK adolescents: A cluster randomised controlled trial," *PLOS Med.*, vol. 17, no. 7, p. e1003210, Jul. 2020, doi: 10.1371/journal.pmed.1003210.

[16] S. A. Costigan, N. D. Ridgers, N. Eather, R. C. Plotnikoff, N. Harris, and D. R. Lubans, "Exploring the impact of high intensity interval training on adolescents' objectively measured physical activity: Findings from a randomized controlled trial," *J. Sports Sci.*, vol. 36, no. 10, pp. 1087–1094, May 2018, doi: 10.1080/02640414.2017.1356026.

[17] A. M. Dennett *et al.*, "Motivational interviewing added to oncology rehabilitation did not improve moderate-intensity physical activity in cancer survivors: a randomised trial," *J. Physiother.*, vol. 64, no. 4, pp. 255–263, Oct. 2018, doi: 10.1016/j.jphys.2018.08.003.

[18] M. Driediger *et al.*, "The Impact of Shorter, More Frequent Outdoor Play Periods on Preschoolers' Physical Activity during Childcare: A Cluster Randomized Controlled Trial," *Int. J. Environ. Res. Public. Health*, vol. 16, no. 21, p. 4126, Nov. 2019, doi: 10.3390/ijerph16214126.

[19] S. M. Edney *et al.*, "A Social Networking and Gamified App to Increase Physical Activity: Cluster RCT," *Am. J. Prev. Med.*, vol. 58, no. 2, pp. e51–e62, Feb. 2020, doi: 10.1016/j.amepre.2019.09.009.

[20] A. Ek *et al.*, "Effectiveness of a 3-Month Mobile Phone–Based Behavior Change Program on Active Transportation and Physical Activity in Adults: Randomized Controlled Trial," *JMIR MHealth UHealth*, vol. 8, no. 6, p. e18531, Jun. 2020, doi: 10.2196/18531.

[21] X. Fischer, J.-N. Kreppke, L. Zahner, M. Gerber, O. Faude, and L. Donath, "Telephone-Based Coaching and Prompting for Physical Activity: Short- and Long-Term Findings of a Randomized Controlled Trial (Movingcall)," *Int. J. Environ. Res. Public. Health*, vol. 16, no. 14, p. 2626, Jul. 2019, doi: 10.3390/ijerph16142626.

[22] P. D. Freitas *et al.*, "A Behavior Change Intervention Aimed at Increasing Physical Activity Improves Clinical Control in Adults With Asthma: A Randomized Controlled Trial," *Chest*, vol. 159, no. 1, pp. 46–57, Jan. 2021, doi: 10.1016/j.chest.2020.08.2113.

[23] Y. Fukuoka, W. Haskell, F. Lin, and E. Vittinghoff, "Short- and Long-term Effects of a Mobile Phone App in Conjunction With Brief In-Person Counseling on Physical Activity Among Physically Inactive Women," *JAMA Netw. Open*, vol. 2, no. 5, p. e194281, May 2019, doi: 10.1001/jamanetworkopen.2019.4281.

[24] O. Galárraga *et al.*, "Small Sustainable Monetary Donation-Based Incentives to Promote Physical Activity: A Randomized Controlled Trial," *Health Psychol. Off. J. Div. Health Psychol. Am. Psychol. Assoc.*, vol. 39, no. 4, pp. 265–268, Apr. 2020, doi: 10.1037/hea0000818.

[25] L. Garcia-Ortiz *et al.*, "Long-Term Effectiveness of a Smartphone App for Improving Healthy Lifestyles in General Population in Primary Care: Randomized Controlled Trial (Evident II Study)," *JMIR MHealth UHealth*, vol. 6, no. 4, p. e107, Apr. 2018, doi: 10.2196/mhealth.9218.

[26] R. H. J. Golsteijn, C. Bolman, E. Volders, D. A. Peels, H. de Vries, and L. Lechner, "Short-term efficacy of a computer-tailored physical activity intervention for prostate and colorectal cancer patients and survivors: a randomized controlled trial," *Int. J. Behav. Nutr. Phys. Act.*, vol. 15, no. 1, p. 106, Oct. 2018, doi: 10.1186/s12966-018-0734-9.

[27] J. M. Guagliano *et al.*, "A whole family-based physical activity promotion intervention: findings from the families reporting every step to health (FRESH) pilot randomised controlled trial," *Int. J. Behav. Nutr. Phys. Act.*, vol. 17, p. 120, Sep. 2020, doi: 10.1186/s12966-020-01025-3.

[28] L. Gutiérrez-Martínez, R. G. Martínez, S. A. González, M. A. Bolívar, O. V. Estupiñan, and O. L. Sarmiento, "Effects of a strategy for the promotion of physical activity in students from Bogotá," *Rev. Saúde Pública*, vol. 52, p. 79, Jul. 2018, doi: 10.11606/S1518-8787.2018052017173.

[29] A. S. Ha, C. Lonsdale, D. R. Lubans, and J. Y. Y. Ng, "Increasing Students' Activity in Physical Education: Results of the Self-determined Exercise and Learning For FITness Trial," *Med. Sci. Sports Exerc.*, vol. 52, no. 3, pp. 696–704, Mar. 2020, doi: 10.1249/MSS.0000000000002172.

[30] K. C. Hamilton, M. T. Richardson, S. McGraw, T. Owens, and J. C. Higginbotham, "A Controlled Evaluation of a CBPR Intervention's Effects on Physical Activity and the Related Psychosocial Constructs Among Minority Children in an Underserved Community," *J. Phys. Act. Health*, vol. 17, no. 1, pp. 37–44, Jan. 2020, doi: 10.1123/jpah.2019-0135.

[31] W. Hardeman *et al.*, "Evaluation of a very brief pedometer-based physical activity intervention delivered in NHS Health Checks in England: The VBI randomised controlled trial," *PLOS Med.*, vol. 17, no. 3, p. e1003046, Mar. 2020, doi: 10.1371/journal.pmed.1003046.

[32] D. M. Harrington *et al.*, "Effectiveness of the 'Girls Active' school-based physical activity programme: A cluster randomised controlled trial," *Int. J. Behav. Nutr. Phys. Act.*, vol. 15, no. 1, p. 40, Apr. 2018, doi: 10.1186/s12966-018-0664-6.

[33] B. Isensee, V. Suchert, J. Hansen, B. Weisser, and R. Hanewinkel, "Effects of a School-Based Pedometer Intervention in Adolescents: 1-Year Follow-Up of a Cluster-Randomized Controlled Trial," *J. Sch. Health*, vol. 88, no. 10, pp. 717–724, 2018, doi: 10.1111/josh.12676.

[34] R. Jago *et al.*, "Action 3:30R: Results of a Cluster Randomised Feasibility Study of a Revised Teaching Assistant-Led Extracurricular Physical Activity Intervention for 8 to 10 Year Olds," *Int. J. Environ. Res. Public. Health*, vol. 16, no. 1, Art. no. 1, Jan. 2019, doi: 10.3390/ijerph16010131.

[35] M. E. Jung *et al.*, "Cardiorespiratory fitness and accelerometer-determined physical activity following one year of free-living high-intensity interval training and moderate-intensity continuous training: a randomized trial," *Int. J. Behav. Nutr. Phys. Act.*, vol. 17, no. 1, p. 25, Feb. 2020, doi: 10.1186/s12966-020-00933-8.

[36] J. W. Kayser *et al.*, "A web-based tailored nursing intervention (TAVIE en m@rche) aimed at increasing walking after an acute coronary syndrome: Multicentre randomized trial," *J. Adv. Nurs.*, vol. 75, no. 11, pp. 2727–2741, 2019, doi: 10.1111/jan.14119.

[37] J. Keller *et al.*, "Long-term effects of a dyadic planning intervention with couples motivated to increase physical activity," *Psychol. Sport Exerc.*, vol. 49, p. 101710, Jul. 2020, doi: 10.1016/j.psychsport.2020.101710.

[38] J. Kernot, L. Lewis, T. Olds, and C. Maher, "Effectiveness of a Facebook-Delivered Physical Activity Intervention for Postpartum Women: A Randomized Controlled Trial," *J. Phys. Act. Health*, vol. 16, no. 2, pp. 125–133, Feb. 2019.

[39] J. Kerr *et al.*, "Cluster randomized controlled trial of a multilevel physical activity intervention for older adults," *Int. J. Behav. Nutr. Phys. Act.*, vol. 15, no. 1, p. 32, Apr. 2018, doi: 10.1186/s12966-018-0658-4.

[40] E. Kolle *et al.*, "The effect of a school-based intervention on physical activity, cardiorespiratory fitness and muscle strength: the School in Motion cluster randomized trial," *Int. J. Behav. Nutr. Phys. Act.*, vol. 17, no. 1, p. 154, Nov. 2020, doi: 10.1186/s12966-020-01060-0.

[41] Y.-P. Lo, S.-L. Chiang, C.-H. Lin, H.-C. Liu, and L.-C. Chiang, "Effects of Individualized Aerobic Exercise Training on Physical Activity and Health-Related Physical Fitness among Middle-Aged and Older Adults with Multimorbidity: A Randomized Controlled Trial," *Int. J. Environ. Res. Public. Health*, vol. 18, no. 1, p. 101, Jan. 2021, doi: 10.3390/ijerph18010101.

[42] J. Looyestyn, J. Kernot, K. Boshoff, and C. Maher, "A Web-Based, Social Networking Beginners' Running Intervention for Adults Aged 18 to 50 Years Delivered via a Facebook Group: Randomized Controlled Trial," *J. Med. Internet Res.*, vol. 20, no. 2, p. e67, Feb. 2018, doi: 10.2196/jmir.7862.

[43] C. Lugones-Sanchez *et al.*, "Effectiveness of an mHealth Intervention Combining a Smartphone App and Smart Band on Body Composition in an Overweight and Obese Population: Randomized Controlled Trial (EVIDENT 3 Study)," *JMIR MHealth UHealth*, vol. 8, no. 11, p. e21771, Nov. 2020, doi: 10.2196/21771.

[44] S. Lundqvist *et al.*, "Long-term physical activity on prescription intervention for patients with insufficient physical activity level—a randomized controlled trial," *Trials*, vol. 21, no. 1, p. 793, Sep. 2020, doi: 10.1186/s13063-020-04727-y.

[45] B. M. Lynch *et al.*, "A randomized controlled trial of a wearable technology-based intervention for increasing moderate to vigorous physical activity and reducing sedentary behavior in breast cancer survivors: The ACTIVATE Trial," *Cancer*, vol. 125, no. 16, pp. 2846–2855, 2019, doi: 10.1002/cncr.32143.

[46] J. K. Ma, C. R. West, and K. A. Martin Ginis, "The Effects of a Patient and Provider Co-Developed, Behavioral Physical Activity Intervention on Physical Activity, Psychosocial Predictors, and Fitness in Individuals with Spinal Cord Injury: A Randomized Controlled Trial," *Sports Med.*, vol. 49, no. 7, pp. 1117–1131, Jul. 2019, doi: 10.1007/s40279-019-01118-5.

[47] S. K. Mama *et al.*, "Feasibility and acceptability of a faith-based mind-body intervention among African American adults," *Transl. Behav. Med.*, vol. 10, no. 4, pp. 928–937, Nov. 2020, doi: 10.1093/tbm/iby114.

[48] M. N. Mascarenhas, J. M. Chan, E. Vittinghoff, E. L. V. Blarigan, and F. Hecht, "Increasing Physical Activity in Mothers Using Video Exercise Groups and Exercise Mobile Apps: Randomized Controlled Trial," *J. Med. Internet Res.*, vol. 20, no. 5, p. e9310, May 2018, doi: 10.2196/jmir.9310.

[49] C. Maxwell-Smith *et al.*, "A randomized controlled trial of WATAAP to promote physical activity in colorectal and endometrial cancer survivors," *Psychooncology.*, vol. 28, no. 7, pp. 1420–1429, Jul. 2019, doi: 10.1002/pon.5090.

[50] J. McNeil *et al.*, "Effects of prescribed aerobic exercise volume on physical activity and sedentary time in postmenopausal women: a randomized controlled trial," *Int. J. Behav. Nutr. Phys. Act.*, vol. 15, no. 1, p. 27, Mar. 2018, doi: 10.1186/s12966-018-0659-3.

[51] J. McNeil *et al.*, "Activity Tracker to Prescribe Various Exercise Intensities in Breast Cancer Survivors," *Med. Sci. Sports Exerc.*, vol. 51, no. 5, pp. 930–940, May 2019, doi: 10.1249/MSS.0000000000001890.

[52] Y. Meng, M. M. Manore, J. M. Schuna, M. M. Patton-Lopez, A. Branscum, and S. S. Wong, "Promoting Healthy Diet, Physical Activity, and Life-Skills in High School Athletes: Results from the WAVE Ripples for Change Childhood Obesity Prevention Two-Year Intervention," *Nutrients*, vol. 10, no. 7, p. 947, Jul. 2018, doi: 10.3390/nu10070947.

[53] B. L. Mitchell *et al.*, "Promoting physical activity in rural Australian adults using an online intervention," *J. Sci. Med. Sport*, vol. 22, no. 1, pp. 70–75, Jan. 2019, doi: 10.1016/j.jsams.2018.07.002.

[54] J. L. Morris *et al.*, "A Pedometer-Based Physically Active Learning Intervention: The Importance of Using Preintervention Physical Activity Categories to Assess Effectiveness," *Pediatr. Exerc. Sci.*, vol. 31, no. 3, pp. 356–362, Aug. 2019, doi: 10.1123/pes.2018-0128.

[55] S. Muellmann *et al.*, "Effects of two web-based interventions promoting physical activity among older adults compared to a delayed intervention control group in Northwestern Germany: Results of the PROMOTE community-based intervention trial," *Prev. Med. Rep.*, vol. 15, p. 100958, Sep. 2019, doi: 10.1016/j.pmedr.2019.100958.

[56] F. Müller-Riemenschneider *et al.*, "Effectiveness of prescribing physical activity in parks to improve health and wellbeing - the park prescription randomized controlled trial," *Int. J. Behav. Nutr. Phys. Act.*, vol. 17, no. 1, p. 42, Mar. 2020, doi: 10.1186/s12966-020-00941-8.

[57] B. Murawski *et al.*, "Efficacy of an m-Health Physical Activity and Sleep Health Intervention for Adults: A Randomized Waitlist-Controlled Trial," *Am. J. Prev. Med.*, vol. 57, no. 4, pp. 503–514, Oct. 2019, doi: 10.1016/j.amepre.2019.05.009.

[58] N. K. Nathan *et al.*, "Implementation of a School Physical Activity Policy Improves Student Physical Activity Levels: Outcomes of a Cluster-Randomized Controlled Trial," *J. Phys. Act. Health*, vol. 17, no. 10, pp. 1009–1018, Sep. 2020, doi: 10.1123/jpah.2019-0595.

[59] C. F. J. Nooijen *et al.*, "The effectiveness of multi-component interventions targeting physical activity or sedentary behaviour amongst office workers: a three-arm cluster randomised controlled trial," *BMC Public Health*, vol. 20, no. 1, p. 1329, Sep. 2020, doi: 10.1186/s12889-020-09433-7.

[60] R. Nourizadeh, S. Azami, A. Farshbaf-Khalili, and E. Mehrabi, "The Effect of Motivational Interviewing on Women with Overweight and Obesity Before Conception," *J. Nutr. Educ. Behav.*, vol. 52, no. 9, pp. 859–866, Sep. 2020, doi: 10.1016/j.jneb.2020.04.219.

[61] A. Ojeda-Rodríguez *et al.*, "Association between favourable changes in objectively measured physical activity and telomere length after a lifestyle intervention in pediatric patients with abdominal obesity," *Appl. Physiol. Nutr. Metab.*, vol. 46, no. 3, pp. 205–212, Mar. 2021, doi: 10.1139/apnm-2020-0297.

[62] C. Ozemek, S. J. Strath, K. Riggin, M. P. Harber, M. T. Imboden, and L. A. Kaminsky, "Pedometer Feedback Interventions Increase Daily Physical Activity in Phase III Cardiac Rehabilitation Participants," *J. Cardiopulm. Rehabil. Prev.*, vol. 40, no. 3, pp. 183–188, May 2020, doi: 10.1097/HCR.0000000000000472.

[63] P. Pallante, C. Perales, V. Rigsby, K. Wilson, and D. Rubin, "Implementation of a pilot parent-focused physical activity program with Latino families in a Head Start program," *Californian J. Health Promot.*, vol. 17, no. 2, Art. no. 2, Dec. 2019, doi: 10.32398/cjhp.v17i2.2286.

[64] S. K. Park, C. H. Bang, and S. H. Lee, "Evaluating the effect of a smartphone app-based self-management program for people with COPD: A randomized controlled trial," *Appl. Nurs. Res.*, vol. 52, p. 151231, Apr. 2020, doi: 10.1016/j.apnr.2020.151231.

[65] C. A. Patten *et al.*, "Feasibility trial of an unsupervised, facility-based exercise programme for depressed outpatients," *Psychol. Health Med.*, vol. 24, no. 3, pp. 320–332, Mar. 2019, doi: 10.1080/13548506.2018.1499944.

[66] M. Plow, M. Finlayson, J. Liu, R. W. Motl, F. Bethoux, and A. Sattar, "Randomized Controlled Trial of a Telephone-Delivered Physical Activity and Fatigue Self-management Interventions in Adults With Multiple Sclerosis," *Arch. Phys. Med. Rehabil.*, vol. 100, no. 11, pp. 2006–2014, Nov. 2019, doi: 10.1016/j.apmr.2019.04.022.

[67] Z. C. Pope, N. Zeng, R. Zhang, H. Y. Lee, and Z. Gao, "Effectiveness of Combined Smartwatch and Social Media Intervention on Breast Cancer Survivor Health Outcomes: A 10-Week Pilot Randomized Trial," *J. Clin. Med.*, vol. 7, no. 6, Art. no. 6, Jun. 2018, doi: 10.3390/jcm7060140.

[68] Z. C. Pope, D. J. Barr-Anderson, B. A. Lewis, M. A. Pereira, and Z. Gao, "Use of Wearable Technology and Social Media to Improve Physical Activity and Dietary Behaviors among College Students: A 12-Week Randomized Pilot Study," *Int. J. Environ. Res. Public. Health*, vol. 16, no. 19, p. 3579, Oct. 2019, doi: 10.3390/ijerph16193579.

[69] L. Poppe *et al.*, "Efficacy of a Self-Regulation–Based Electronic and Mobile Health Intervention Targeting an Active Lifestyle in Adults Having Type 2 Diabetes and in Adults Aged 50 Years or Older: Two Randomized Controlled Trials," *J. Med. Internet Res.*, vol. 21, no. 8, p. e13363, Aug. 2019, doi: 10.2196/13363.

[70] G. Prado *et al.*, “Results of a Family-Based Intervention Promoting Healthy Weight Strategies in Overweight Hispanic Adolescents and Parents: An RCT,” *Am. J. Prev. Med.*, vol. 59, no. 5, pp. 658–668, Nov. 2020, doi: 10.1016/j.amepre.2020.06.010.

[71] J. L. Reed *et al.*, “The Impact of Web-Based Feedback on Physical Activity and Cardiovascular Health of Nurses Working in a Cardiovascular Setting: A Randomized Trial,” *Front. Physiol.*, vol. 9, p. 142, 2018, doi: 10.3389/fphys.2018.00142.

[72] S. E. Reedman, R. N. Boyd, S. G. Trost, C. Elliott, and L. Sakzewski, “Efficacy of Participation-Focused Therapy on Performance of Physical Activity Participation Goals and Habitual Physical Activity in Children With Cerebral Palsy: A Randomized Controlled Trial,” *Arch. Phys. Med. Rehabil.*, vol. 100, no. 4, pp. 676–686, Apr. 2019, doi: 10.1016/j.apmr.2018.11.012.

[73] B. Reich *et al.*, “Effects of active commuting on cardiovascular risk factors: GISMO—a randomized controlled feasibility study,” *Scand. J. Med. Sci. Sports*, vol. 30, no. S1, pp. 15–23, 2020, doi: 10.1111/sms.13697.

[74] R. D. Reid *et al.*, “A Randomized Controlled Trial of an Exercise Maintenance Intervention in Men and Women After Cardiac Rehabilitation (ECO-PCR Trial),” *Can. J. Cardiol.*, vol. 37, no. 5, pp. 794–802, May 2021, doi: 10.1016/j.cjca.2020.10.015.

[75] K. Riiser, K. R. Richardsen, A. L. H. Haugen, S. Lund, and K. Løndal, “Active play in ASP –a matched-pair cluster-randomized trial investigating the effectiveness of an intervention in after-school programs for supporting children’s physical activity,” *BMC Public Health*, vol. 20, no. 1, p. 500, Apr. 2020, doi: 10.1186/s12889-020-08645-1.

[76] L. B. Robbins *et al.*, “Intervention Effects of ‘Girls on the Move’ on Increasing Physical Activity: A Group Randomized Trial,” *Ann. Behav. Med. Publ. Soc. Behav. Med.*, vol. 53, no. 5, pp. 493–500, 2019, doi: 10.1093/abm/kay054.

[77] S. A. Robinson, A. N. Bisson, M. L. Hughes, J. Ebert, and M. E. Lachman, “Time for Change: Using Implementation Intentions to Promote Physical Activity in a Randomised Pilot Trial,” *Psychol. Health*, vol. 34, no. 2, pp. 232–254, Feb. 2019, doi: 10.1080/08870446.2018.1539487.

[78] M. G. Rodriguez-Hernandez and D. W. Wadsworth, “The effect of 2 walking programs on aerobic fitness, body composition, and physical activity in sedentary office employees,” *PLOS ONE*, vol. 14, no. 1, p. e0210447, Jan. 2019, doi: 10.1371/journal.pone.0210447.

[79] M. E. Rollo *et al.*, “The Feasibility and Preliminary Efficacy of an eHealth Lifestyle Program in Women with Recent Gestational Diabetes Mellitus: A Pilot Study,” *Int. J. Environ. Res. Public. Health*, vol. 17, no. 19, p. 7115, Oct. 2020, doi: 10.3390/ijerph17197115.

[80] G. R. Ruissen, R. E. Rhodes, P. R. E. Crocker, and M. R. Beauchamp, “Affective mental contrasting to enhance physical activity: A randomized controlled trial,” *Health Psychol.*, vol. 37, no. 1, pp. 51–60, 2018, doi: 10.1037/hea0000551.

[81] M. Sala, B. Geary, and A. S. Baldwin, “A Mindfulness-Based Physical Activity Intervention: A Randomized Pilot Study,” *Psychosom. Med.*, vol. 83, no. 6, pp. 615–623, Aug. 2021, doi: 10.1097/PSY.0000000000000885.

[82] A. M. Sánchez-López, M. J. Menor-Rodríguez, J. C. Sánchez-García, and M. J. Aguilar-Cordero, “Play as a Method to Reduce Overweight and Obesity in Children: An RCT,” *Int. J. Environ. Res. Public. Health*, vol. 17, no. 1, p. 346, Jan. 2020, doi: 10.3390/ijerph17010346.

[83] V. O. A. Santos *et al.*, “Effects of High-Intensity Interval and Moderate-Intensity Continuous Exercise on Physical Activity and Sedentary Behavior Levels in Inactive Obese Males: A Crossover Trial,” *J. Sports Sci. Med.*, vol. 18, no. 3, pp. 390–398, Aug. 2019.

[84] S. J. Sebire *et al.*, “Results of a feasibility cluster randomised controlled trial of a peer-led school-based intervention to increase the physical activity of adolescent girls (PLAN-A),” *Int. J. Behav. Nutr. Phys. Act.*, vol. 15, no. 1, p. 50, Jun. 2018, doi: 10.1186/s12966-018-0682-4.

[85] P. H. Seljebotn, I. Skage, A. Riskedal, M. Olsen, S. E. Kvalø, and S. M. Dyrstad, “Physically active academic lessons and effect on physical activity and aerobic fitness. The Active School study: A cluster randomized controlled trial,” *Prev. Med. Rep.*, vol. 13, pp. 183–188, 2019, doi: 10.1016/j.pmedr.2018.12.009.

[86] B. Singh, R. R. Spence, C. X. Sandler, J. Tanner, and S. C. Hayes, "Feasibility and effect of a physical activity counselling session with or without provision of an activity tracker on maintenance of physical activity in women with breast cancer - A randomised controlled trial," *J. Sci. Med. Sport*, vol. 23, no. 3, pp. 283–290, Mar. 2020, doi: 10.1016/j.jsams.2019.09.019.

[87] B. Spring *et al.*, "Multicomponent mHealth Intervention for Large, Sustained Change in Multiple Diet and Activity Risk Behaviors: The Make Better Choices 2 Randomized Controlled Trial," *J. Med. Internet Res.*, vol. 20, no. 6, p. e10528, Jun. 2018, doi: 10.2196/10528.

[88] A. Taylor *et al.*, "Randomised controlled trial of an augmented exercise referral scheme using web-based behavioural support for inactive adults with chronic health conditions: the e-coachER trial," *Br. J. Sports Med.*, vol. 55, no. 8, pp. 444–450, 2020, doi: 10.1136/bjsports-2020-103121.

[89] C. Tudor-Locke *et al.*, "Evaluation of Step-Counting Interventions Differing on Intensity Messages," *J. Phys. Act. Health*, vol. 17, no. 1, pp. 21–28, Jan. 2020, doi: 10.1123/jpah.2018-0439.

[90] K. Uemura, M. Yamada, and H. Okamoto, "The Effectiveness of an Active Learning Program in Promoting a Healthy Lifestyle among Older Adults with Low Health Literacy: A Randomized Controlled Trial," *Gerontology*, vol. 67, no. 1, pp. 25–35, 2021, doi: 10.1159/000511357.

[91] V. van den Berg, A. S. Singh, A. Komen, C. Hazelebach, I. van Hilvoorde, and M. J. M. Chinapaw, "Integrating Juggling with Math Lessons: A Randomized Controlled Trial Assessing Effects of Physically Active Learning on Maths Performance and Enjoyment in Primary School Children," *Int. J. Environ. Res. Public. Health*, vol. 16, no. 14, p. 2452, Jul. 2019, doi: 10.3390/ijerph16142452.

[92] D. Van Dyck, K. Herman, L. Poppe, G. Crombez, I. De Bourdeaudhuij, and F. Gheysen, "Results of MyPlan 2.0 on Physical Activity in Older Belgian Adults: Randomized Controlled Trial," *J. Med. Internet Res.*, vol. 21, no. 10, p. e13219, Oct. 2019, doi: 10.2196/13219.

[93] K. Van Hoye, A. I. Wijtzes, J. Lefevre, S. De Baere, and F. Boen, "Year-round effects of a four-week randomized controlled trial using different types of feedback on employees' physical activity," *BMC Public Health*, vol. 18, p. 492, Apr. 2018, doi: 10.1186/s12889-018-5402-0.

[94] C. Vandelanotte *et al.*, "Are web-based personally tailored physical activity videos more effective than personally tailored text-based interventions? Results from the three-arm randomised controlled TaylorActive trial," *Br. J. Sports Med.*, vol. 55, no. 6, pp. 336–343, Mar. 2021, doi: 10.1136/bjsports-2020-102521.

[95] S. L. C. Veldman *et al.*, "Promoting Physical Activity and Executive Functions Among Children: A Cluster Randomized Controlled Trial of an After-School Program in Australia," *J. Phys. Act. Health*, vol. 17, no. 10, pp. 940–946, Aug. 2020, doi: 10.1123/jpah.2019-0381.

[96] L. Viester, E. A. L. M. Verhagen, P. M. Bongers, and A. J. van der Beek, "Effectiveness of a Worksite Intervention for Male Construction Workers on Dietary and Physical Activity Behaviors, Body Mass Index, and Health Outcomes: Results of a Randomized Controlled Trial," *Am. J. Health Promot. AJHP*, vol. 32, no. 3, pp. 795–805, Mar. 2018, doi: 10.1177/0890117117694450.

[97] E. Volders, C. A. W. Bolman, R. H. M. de Groot, P. Verboon, and L. Lechner, "The Effect of Active Plus, a Computer-Tailored Physical Activity Intervention, on the Physical Activity of Older Adults with Chronic Illness(es)—A Cluster Randomized Controlled Trial," *Int. J. Environ. Res. Public. Health*, vol. 17, no. 7, p. 2590, Apr. 2020, doi: 10.3390/ijerph17072590.

[98] D. D. Wadsworth, J. L. Johnson, A. V. Carroll, M. M. Pangelinan, M. E. Rudisill, and J. Sassi, "Intervention Strategies to Elicit MVPA in Preschoolers during Outdoor Play," *Int. J. Environ. Res. Public. Health*, vol. 17, no. 2, Art. no. 2, Jan. 2020, doi: 10.3390/ijerph17020650.

[99] Z. Xu *et al.*, "A Mobile-Based Intervention for Dietary Behavior and Physical Activity Change in Individuals at High Risk for Type 2 Diabetes Mellitus: Randomized Controlled Trial," *JMIR MHealth UHealth*, vol. 8, no. 11, p. e19869, Nov. 2020, doi: 10.2196/19869.

[100] S. L. Yoong *et al.*, "A pilot randomized controlled trial examining the impact of a sleep intervention targeting home routines on young children's (3–6 years) physical activity," *Pediatr. Obes.*, vol. 14, no. 4, p. e12481, 2019, doi: 10.1111/ijpo.12481.
